# Attitude of Asian Parkinson Patients towards Clinical Research and Tissue Donation

**DOI:** 10.1155/2020/2542756

**Published:** 2020-02-19

**Authors:** Shazma Khan, Joel Y. J. Foo, Nicole S. Y. Chia, Sherwin J. U. Agustin, Shermyn X. M. Neo, Kay-Yaw Tay, Wing-Lok Au, Adeline S. L. Ng, Louis C. S. Tan

**Affiliations:** ^1^Department of Neurology, National Neuroscience Institute, Singapore; ^2^Parkinson Disease and Movement Disorders Centre, Parkinson Foundation Center of Excellence, National Neuroscience Institute, Singapore; ^3^Department of Research, National Neuroscience Institute, Singapore

## Abstract

**Objective:**

The success of clinical research and tissue donation programs are highly dependent on recruitment of willing volunteers. A comprehensive survey of patient preferences and attitudes can help identify and address barriers hindering the recruitment for research.

**Method:**

This is a cross-sectional study on 105 Parkinson's disease patients who completed an interviewer-administered questionnaire.

**Results:**

Out of 105 respondents, 48% of patients had either already participated in clinical research or were keen to participate. About 80% believed clinical research to be safe for their health and privacy. More than 70% of participants were willing to donate blood, urine, or stool, while 16% were agreeable for cerebrospinal fluid sample donation. Motivating factors for clinical research included altruism (64%) and contribution to advance medical knowledge (64%). Common reasons for unwillingness towards clinical research included the risks involved (43%), time constraints (33%), and mobility challenges (24%).

**Conclusion:**

The attitude of Singaporean Parkinson patients toward clinical research and tissue donation is encouraging with about half of the participants willing to support clinical research. Three-quarters of patients would support tissue donations. Participation in research may be further increased with greater patient and public education to overcome misconceptions and also by limiting the demands of studies.

## 1. Introduction

Clinical research and tissue donation play an integral role in advancing medical knowledge and hence improving healthcare in any country. These components are especially crucial in the study of neurodegenerative conditions, which remain poorly understood with regard to diagnosis and curative treatment. Both clinical research and tissue donation have their success rates highly dependent on the design of ethically sound and effective recruitment of willing volunteers. This continues to be a prevalent challenge against advancement of medical knowledge through research. Recently observed trends have depicted low rates of participation in clinical research and tissue donation, especially amongst Asian populations [1–3].

Often cited reasons for not participating in research studies include (1) inconvenience and discomfort, (2) lack of trust on trial medicines, treating doctors, or hospital facilities, (3) lack of information about research, (4) family's refusal to allow participation, and (5) uncertainty or fear of the unknown [4, 5]. Asian countries such as Oman, Saudi Arabia, and India showed interest rates of 59%, 30%, and 46%, respectively, for participation in clinical research [1, 2, 6]. A comprehensive review by Wai et al. has summarized the findings of 18 different studies that evaluated experiences of patients regarding their prior research participation and the influence of that on consenting to reuse of leftover tissues for future research. The study highlighted the importance of a transparent consent process and protection of patient privacy, as well as safeguard of their donated tissue from unethical or commercial use. The study highlighted the importance of ethical conduct of research projects [7].

This study is designed to evaluate Singaporean Parkinson's disease (PD) patients' knowledge and perception of clinical research and tissue donation, their willingness to participate, and the reasons for reluctance to participate in research studies. By identifying the barriers hindering the research advancement, this study will help improve future research study designs and recruitment procedures by understanding patient perspectives and developing ethically sound protocols to increase participant satisfaction.

## 2. Methods

### 2.1. Participants

This is a cross-sectional study of 105 patients who presented to the neurology clinics of a tertiary care hospital in Singapore. The study was approved by the SingHealth Centralized Institutional Review Board. The study included patients who had been diagnosed with PD and who were able to converse in English or Mandarin. Only patients who were not demented and cognitively able to undertake the survey as assessed by their treating physicians were invited to take part in the study. After being screened for eligibility by their primary physician, participants were seated in a quiet area to answer a questionnaire administered by a trained research coordinator that was completed in one sitting of 15–20 minutes.

### 2.2. Data Collection

The 30-item questionnaire collected information on (1) personal demographics such as age, gender, educational status, ethnicity, religion, and clinical characteristics of disease such as duration, (2) attitudes and beliefs of patients regarding participation in (i) clinical research and (ii) studies involving collection of blood, urine/stool and cerebrospinal fluid (CSF) collection, and (3) reasons of their particular beliefs towards these studies. Standardized explanations on what clinical research and tissue donations entailed were presented prior to the respective questions. Participants were briefed that clinical research referred to both clinical studies and clinical trials. To the questions regarding participation, the participants were required to pick the best response from “strongly agree, agree, neutral/not sure, disagree, and strongly disagree.” Answers pertaining to reasons for their beliefs were selected from a predesigned list of responses. Participants were allowed to give additional reasons that were not found on the list. HY staging of all participants was assessed by their physicians on the day of study participation. For the purpose of brevity, wordings in tables are summarized.

### 2.3. Statistical Analysis

Descriptive statistical analysis was performed and expressed in mean (±standard deviation) or percentage. Multinomial logistic regression was conducted to examine the types of demographic and clinical characteristics associated with willingness to participate in clinical research and tissue donation. A *p* value of <0.05 was considered to indicate statistical significance. The IBM SPSS version 20.0 (IBM Corp, Armonk, NY, USA) was used to perform the analysis.

## 3. Results

### 3.1. Demographics

The survey was completed by 105 PD patients (Table 1). Participants had a mean age of 67.5 (±8.5) years. Most participants were male (55%) and of Chinese ethnicity (84%). Religious affiliations were found among 87% of participants. Average formal education among participants was 10 (±4) years, and the PD duration averaged 9.5 (±5.3) years.

### 3.2. Perception and Attitude towards Clinical Research

Of 105 PD participants, 48% were keen to participate in clinical research despite understanding the possible risks involved (Table 2), with younger patients being more willing to do so (OR = 1.082, *p*= 0.006). Frequently cited factors for willingness to participate in clinical research included desire to advance medical knowledge (64%), benefit other patients (64%), and the belief that participants would receive better care as a result of closer observation (59%) (Table 3). Conversely, the perception of high risks involved (43%), not having enough time (33%), and having mobility issues (24%) were frequently stated deterrents of clinical research participation (Table 4).

The importance of reporting results of clinical research was particularly supported by participants with shorter disease duration (OR = 1.361, *p*=0.024), and the belief that results from past research played a significant role in developing current treatment options resonated more amongst participants who were younger (OR = 1.151, *p*=0.002) or had more years of education (OR = 1.209, *p*=0.026).

There was no significant influence of gender, marital status, ethnicity, or religious affiliation on participation in clinical research.

### 3.3. Perception and Attitude towards Blood, Urine, Stool, and CSF Donation

The majority of respondents was agreeable to donate their urine and stool samples for research (71%). The remaining 29% were dissuaded by key reasons such as an inconvenient collection process (72%), difficulty in urination and defecation (31%), and time constraints (24%). A similar majority were also willing to have their blood sampled for research (72%). Female participants in particular were found to be less agreeable (OR = 6.898, *p*= 0.042) to enroll in research that requires blood collection. Significant reasons for the unwillingness included having fear of side effects (52%) and needle pricks (41%). A number of patients also believed the blood collection process to be inconvenient for them (37%). Forty-six percent participants were agreeable to donate blood or other body tissues for genetic testing even if the test results were not to be disclosed. In contrast to urine, stool, and blood sample donation, respondents were not as keen to take part in clinical research involving cerebrospinal fluid (CSF) collection, with the positive response rate being 16% only. Major perceptions against CSF collection revolved around the invasiveness of the procedure (74%) and the risk of potential side effects such as pain, bleeding, nerve injury, and headache (63%).

## 4. Discussion

As researchers are faced with the problem of patient participation in research globally, understanding the barriers limiting patients' interests in research is important in designing future research projects that ensure participant satisfaction and recruitment. While extensive work has been done to evaluate Western patients' perspectives, there have been only few studies representing Asian patients' attitude towards research participation, which could be due to lack of patient or physician interest as well as limited funding and infrastructure to promote research [8, 9], while various Asian studies have been performed to assess attitudes of healthy subjects or mixed group of patients towards research participation [2, 6, 10–14]; to the best of our knowledge, this is the first Asian study on Parkinson patients to understand their attitudes towards clinical research and tissue donation.

Despite understanding the possible risks involved, our study revealed that 48% of patients were willing to participate in clinical research, with an even higher number of patients willing to contribute blood (72%) or urine/stool (71%). As opposed to the Omani, Saudi, and Indian patients, the Singaporean population was found to be better aware and educated about research [1, 2, 6, 10]. Participants understood the importance of research in development of current medical knowledge, and majority trusted medical facilities in guarding their safety and privacy, whereas previous Asian studies have identified a general mistrust towards government and medical facilities in conducting ethical research and protecting patient confidentiality [7, 11]. This trust on the physicians and healthcare facilities is the major contributing factor for the positive response of Singaporean population towards research participation.

The motivation for clinical research participation included both altruistic as well as nonaltruistic factors. The desire to help other patients with similar disease and to help advance medical knowledge were found to be the main reasons for participation which are consistent with the international literature [5]. The primary personal reasons for participation were the belief that participants would have closer and more careful monitoring and receive the best available medical treatment by being part of clinical research.

Forty-three percent of our participants believed that participation in clinical research was too risky for them compared to 80% of Saudi Arabian and 48% of Indian participants [2, 10]. As the patients in our study did not particularly mention the risks they feared, these possibly could be the fear of unknown or of potential side effects. Confidentiality breach was not found to be a major concern as opposed to other studies where misuse of information and doubts regarding randomization were predominant reasons for nonparticipation [7, 11, 13, 15].

Time constraint was another reason for declining participation. A previous study showed that time constraint was more of a concern to younger participants due to work commitments [8]. Although the majority of our study population was above 65, this reason was voiced by about one-third of the participants. It is possible that due to the interdependent living style among Asians, the aged PD patients are usually accompanied by their children during hospital visits, who have to take time off work to accompany their parents to hospital, and it may be difficult for them to spare extra time for research participation. Hence, it is important to simplify the study design and keep the demands of study precise and pertinent to the research purpose. The flexibility of follow-up visits to suit participants' schedule and reducing the inconvenience, such as by performing home study visits or internet-based evaluations, especially amidst a globally aging population, may increase clinical research participation.

We found that younger participants were more willing to participate in clinical research. This finding is consistent with results from another study, which could be attributed to younger participants being more educated and understanding toward the importance of clinical research [16]. They also believed that the current knowledge and treatment of the disease was the result of past research. It is noteworthy that 46% of our study population were agreeable to donate blood or tissue for genetic testing even if the results were not to be revealed to them which are higher than the 32–37% response rates of American population as per literature [17, 18]. However, patients with shorter disease duration were keen to know the results of the clinical research that they participated in. This could be attributed to their concerns about the disease and its progression as well as their hope that better treatment strategies could be discovered in their lifetime. Many studies done previously in this regard highlighted the patients' desire of wanting to know the results of the studies they participated in [1, 8, 17, 18]. A study by Christopher et al. showed that the nonpublication of trial results goes against the patients' motive of altruism or helping advance medical knowledge [17]. Hence, it is important to share the summarized findings of clinical research with the participants [8, 19]. An even better way is to translate the research findings into clinical implications so as to help the participants understand the importance of their contribution. This is also likely to increase satisfaction from research participation.

While there are many studies regarding organ donation for transplantation, there are barely any studies from Asia pertaining to the attitude of patients towards tissue donation for research purpose. Our study identified positive responses of PD participants towards donation of body tissues such as blood and urine/stool for the purpose of clinical research, with about two-thirds of the participants agreeable for such donations. As opposed to blood and urine/stool donation, the majority was reluctant to consider CSF donation with the major reason being fear of side effects of the invasive procedure. This suggests a lack of patient understanding regarding the safety of CSF tap and raises a need for medical staff to be well aware of and be able to address the misconceptions and fears while approaching patients for tissue donation for the research purpose.

Patients and general public education can play a considerable role in enhancing their knowledge further, spreading awareness about available ongoing or upcoming research, addressing participant concerns, and rectifying their misconceptions. A more effective way is to involve the participants of prior research studies to share their experiences with other patients and public to ameliorate their fears.

More than three-quarter of the participants in our study expected their treating doctors to inform them of the available clinical research options pertinent to their illness, which is in accordance with literature [1, 18]. Patients are also more likely to take part if invited by their trusted physicians [6]. A few studies have emphasized on a lack of physician interest in discussing research participation options with their patients and a very low rate of referral of their patients to clinical research or trials [8]. The reported reasons for not doing so were mainly insufficient information about ongoing research trials, lack of time and resources to confidently discuss with their patients, and being uncertain about where to refer their patients [9]. It is important for doctors and nurse clinicians to build effective communication with their patients to help alleviate fears relating to participation. Although it is recommended that physicians should proactively initiate research recruitment discussions with their patients, they have to remain watchful about the possibility of undue compulsion, which can lead to influence bias [20].

Another factor that may improve participation in research is the concept of providing compensations to the participants. This widely practiced method to enhance enrollment in research may include free clinic visits, free laboratory tests related to research, transport compensations, complimentary parking spaces, gift cards, or cheques [8, 18, 21]. Two studies from India emphasized poor patient knowledge about their research participation rights as only one-third of the patients were aware of the concept of compensations offered to participants [6, 11]. While the idea of financial benefit may not be attractive to some whose main aim is altruism, it could strongly influence others to participate [2, 7].

This study is conducted on the multiracial Singapore PD population, and the results of this study may serve as the basis to improve the design of future research projects. However, this single center study should be followed by larger multicenter national and international studies for more generalized results. Also, including healthy subjects as a control group in the study would have strengthened the results and highlighted the differences in attitudes of PD patients as compared with healthy subjects for research participation. The limitations of an individual interview structure used in this study have to be acknowledged. Questions worth further exploration include the reasons for unwillingness to participate in research. Future studies that aim to capture similar perceptions may wish to adopt a focus group format that is more likely to disclose more sensitive and personal views. Another way to better understand participant perspectives is to use open-ended questionnaires that can allow the participants to express their beliefs openly without getting influenced by predesigned responses [22].

The inability to recruit an adequate number of participants for clinical research and tissue donation affects the quality of research. Based on our study, the responsiveness of Singaporean PD patients towards clinical research and tissue donation seems encouraging. However, researchers need to understand that patient willingness for research participation is based on complex, multifactorial reasons. It is important to make information regarding clinical research and tissue donation projects easily available to physicians and the public. Patient and public education by doctors, nurses, and previous research participants will help overcome misconceptions and hence improve participation.

## Figures and Tables

**Table 1:** Demographic and clinical characteristics of survey participants (*n* = 105).

Variable	Mean ± SD or *n* (%)
Age (y)	67.5 ± 8.5
Male	58 (55)
Ethnic groups	
Chinese	88 (84)
Non-Chinese (Malay, Indian, and others)	17 (16)
Religious affiliation	
Christian	42 (40)
Buddhist	32 (30)
Muslim	8 (8)
Hindu	5 (5)
Taoist	4 (4)
No affiliation	14 (13)
Marital status	
Married/living with a partner	82 (78)
Widowed/divorced	11 (11)
Never married	12 (11)
Housing type	
Landed property	15 (14)
Condominium	12 (11)
5-room flat	22 (21)
4-room flat	35 (33)
3-room flat	18 (17)
1/2-room flat	2 (2)
Education (y)	10.0 ± 4.0
Parkinson's disease duration (y)	9.5 ± 5.3
Hoehn and Yahr stage	2.4 ± 0.8
Charlson comorbidity index	2.6 ± 1.2

**Table 2:** Positive responses to questions assessing patient's attitudes and preferences towards participation in clinical research ^*∗*^(*n* = 105).

Question	*n* (%)^†^
(1) Have you been asked to participate in clinical research?	
(i) If yes, did you agree to participate in that clinical research? (*n* = 40)	22 (55)
(ii) If no, would you be interested to participate in clinical research related to your neurological condition? (*n* = 65)	29 (45)
(2) I am keen on participating in clinical research despite understanding that the prescribed treatment may have a small risk of possible side effects.	50 (48)
(3) It is important for the investigator to inform me about the results from a clinical research which I have participated in.	88 (84)
(4) Results of clinical research are still worth reporting even if the treatments were seen to be ineffective.	87 (83)
(5) My treating physician should inform me about any clinical research of interest.	81 (77)
(6) Most organizations conducting clinical research have taken all reasonable precautions to ensure participant's safety.	83 (79)
(7) Most organizations conducting clinical research have taken all reasonable precautions to safeguard participant's privacy.	85 (81)
(8) Most of the current treatments in medicine are a result of evidence gained from past clinical research.	75 (71)
(9) Conducting clinical research help medical professionals treat and understand my medical condition better.	86 (82)

^∗^Positive responses were “yes” to “yes/no” questions and “agree” or “strongly agree” to questions with Likert scale responses ranging from 1 to 5 as follows: (1) strongly agree, (2) agree, (3) neutral, (4) disagree, and (5) strongly disagree. ^†^*n* = number (percentage).

**Table 3:** Reasons of willingness to participate in clinical research ^*∗*^(*n* = 64).

Reason	*n* (%)^†^
(1) I would like to advance medical knowledge of my condition.	40 (63)
(2) I would like to benefit other patients in future.	40 (63)
(3) I believe I will be monitored more closely and receive better care as part of the research.	38 (59)
(4) I thought the trial offered the best treatment available.	30 (47)
(5) I trust the doctor who is treating me.	14 (22)
(6) I think my condition will get worse unless I take part in the research.	12 (19)
(7) My family was keen for me to participate.	4 (6)

^*∗*^Sample size includes patients with positive responses as well as those who answered “neutral” or “not sure” in the corresponding base question. ^†^*n* = number (percentage).

**Table 4:** Reasons of unwillingness to participate in clinical research ^*∗*^(*n* = 54).

Reason	*n* (%)^†^
(1) I feel that the risk of participating is too high.	23 (43)
(2) I do not have time to participate.	18 (33)
(3) I feel my immobility will make participation difficult.	13 (24)
(4) My privacy might be lost as a result of the clinical research.	4 (7)
(5) My family was not keen for me to participate.	3 (6)
(6) I believe it will not help improve my medical condition.	3 (6)
(7) It is too complicated of a process.	3 (4)
(8) I am skeptical about research and its benefits.	2 (4)
(9) I have a conservative mindset.	2 (4)
(10) I have no one to accompany me to the venue.	2 (4)
(11) I am unable to think of any reasons.	2 (4)
(12) I have a fear of procedures involving injections/blood collection.	1 (2)
(13) I have a fear of procedures done in confined places.	1 (2)
(14) I do not feel comfortable participating.	1 (2)
(15) I feel that participating in clinical trials is secondary to coming to terms with my condition.	1 (2)
(16) I have no interest in these activities and prefer to stay at home.	1 (2)

^*∗*^Sample size includes patients with negative responses as well as those who answered “neutral” or “not sure” in the corresponding base question. ^†^*n* = number (percentage).

## Data Availability

The questionnaire used to support the findings of this study is available from the corresponding author upon request.
